# MoS_2_ and MoS_2_ Nanocomposites for Adsorption and Photodegradation of Water Pollutants: A Review

**DOI:** 10.3390/molecules27206782

**Published:** 2022-10-11

**Authors:** Leonardo O. Amaral, Ana L. Daniel-da-Silva

**Affiliations:** Department of Chemistry and CICECO-Aveiro Institute of Materials, University of Aveiro, 3810-193 Aveiro, Portugal

**Keywords:** molybdenum disulfide, nanocomposites, adsorption, photocatalysis, organic contaminants, heavy metal ions

## Abstract

The need for fresh and conveniently treated water has become a major concern in recent years. Molybdenum disulfide (MoS_2_) nanomaterials are attracting attention in various fields, such as energy, hydrogen production, and water decontamination. This review provides an overview of the recent developments in MoS_2_-based nanomaterials for water treatment via adsorption and photodegradation. Primary attention is given to the structure, properties, and major methods for the synthesis and modification of MoS_2_, aiming for efficient water-contaminant removal. The combination of MoS_2_ with other components results in nanocomposites that can be separated easily or that present enhanced adsorptive and photocatalytic properties. The performance of these materials in the adsorption of heavy metal ions and organic contaminants, such as dyes and drugs, is reviewed. The review also summarizes current progress in the photocatalytic degradation of various water pollutants, using MoS_2_-based nanomaterials under UV-VIS light irradiation. MoS_2_-based materials showed good activity after several reuse cycles and in real water scenarios. Regarding the ecotoxicity of the MoS_2_, the number of studies is still limited, and more work is needed to effectively evaluate the risks of using this nanomaterial in water treatment.

## 1. Introduction

The world’s population has been growing yearly, and an increased need for resources has accompanied this growth [1]. Industrial, agricultural, and medical development have evolved to supply this population growth [1,2]. However, after this substantial increase in resource consumption, pollution had also increased, becoming a huge concern, along with the need for freshwater sources or for effective water treatment [1,3]. In this regard, several approaches for water treatment have been developed, such as adsorption and photocatalysis, to remove pollutants and other threats to human health and ecosystems, such as heavy metals, dyes, drugs, and pesticides, among others [4,5,6,7,8].

Along with the advances in environmental research, in the past few years, several authors have suggested nanomaterials to enhance primary and advanced water treatment [9]. Due to its small size and, consequently, high surface and reactive areas, the use of nanomaterials enables the improvement of several properties compared to bulk material [9]. More recently, two-dimensional (2D) nanomaterials have gained importance. In this group are included materials such as graphene, hexagonal boron nitride, 2D honeycomb silicon, and transition metal dichalcogenides (TMD) such as molybdenum disulfide (MoS_2_) and tungsten disulfide (WS_2_) [10]. TMD are hexagonal, structured and layered materials with the molecular formula MX_2_, where M is a transition metal (Mo, W, or Nb) and X represents a chalcogen element (S, Se, or Te) [11,12]. Within this group of materials, nano-MoS_2_ has gained attention year after year due to its outstanding chemical, electronic, catalytic, optical, and mechanical properties, making it suitable for various fields such as pollution remediation and sensors, medicine, mechanics and electronics, and energy conversion and storage [13,14,15,16,17,18,19,20,21,22,23,24]. When compared to other sulfides, such as zinc sulfide (ZnS), cadmium sulfide (CdS), and tungsten sulfide (WS_2_), MoS_2_ show promising photocatalytic activity, allied to its easy preparation and unique optical and electronic properties [25]. The wide band gaps of ZnS and MnS render them responsive to only UV light; further modification is required to extend the light absorption range [26]. In contrast, MoS_2_ is active with visible light. As a photocatalytic material, MoS_2_ presents a suitable surface area with a large number of active site edges, a layered structure that allows good adsorption capacity, and a tunable band gap between 1.2 eV and 1.9 eV, depending on the number of layers [27]. Regarding safety considerations, Mo is less hazardous and can be used to substitute Cd, which is known to be a human carcinogen.

In this review, we first summarize the main structures and properties of MoS_2_ and the most common methods by which to prepare MoS_2_-based materials. We then critically review the recent advances in MoS_2_-based materials for water treatment and purification through adsorption and photocatalysis.

## 2. MoS_2_ Structure and Properties

In nature, MoS_2_ exists in molybdenite bulk mineral form as a black powder [28]. Bulk MoS_2_ can be exfoliated, obtaining few-layer nanosheets (FLMoS_2_) and single-layer nanosheets (SLMoS_2_) [29,30]. SLMoS_2_ compared to bulk MoS_2_ has intrinsic higher electronic conductivity and presents a direct gap of 1.7–1.9 eV, while bulk MoS_2_ presents an indirect band gap of 1.1–1.3 eV [31,32,33,34]. This relatively small band gap of SLMoS_2_ enables the use of visible light to perform photocatalytic reactions [35,36]. While absent in the bulk material, SLMoS_2_ also shows strong photoluminescence [37]. At room temperature, SLMoS_2_ exhibits an on/off current ratio of 10^8^ and an electron mobility of 200 cm^2^ (V.s)^−1^ [38]. Notably, several properties of MoS_2_ can be adjusted by varying the number of layers and polymorphism. SLMoS_2_ presents a sandwich structure with a thickness of 0.6 nm, composed of two layers of atomic S, with a single layer of atomic Mo between, linked through Mo-S covalent bonds [39,40,41]. In the case of FLMoS_2_, the different sheets interact with each other via weak van der Waals (vdW) forces [40,42]. Because of these weak vdW forces, changing SLMoS_2_ to FLMoS_2_ and gaining the reverse reaction is easily achieved [41]. Kumar and Mishra demonstrated, via molecular simulation, the mechanism of solvent-assisted exfoliation to turn bulk MoS_2_ into FLMoS_2_ by applying a shock-wave method [43].

**Figure 1:**
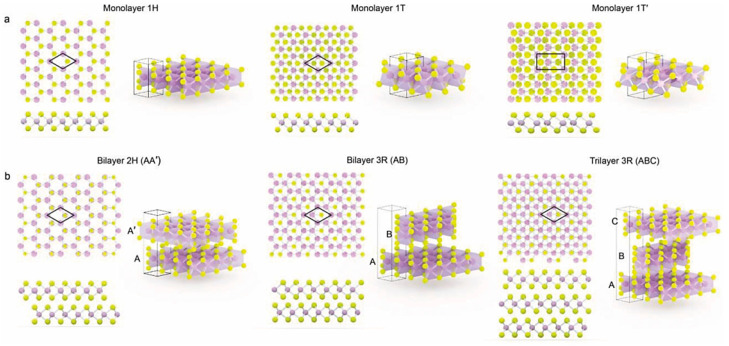
Atomic structures of various polymorphs of MoS_2_. (**a**) Monolayer structures; (**b**) bilayer and trilayer structures (yellow—S atoms; purple—Mo atoms). A, A’, B and C represent S-Mo-S layers. AA’, AB and ABC represent the stacking order sequence Reproduced from [44], with permission from John Wiley & Sons, Copyright 2018 Wiley–VCH GmbH.

MoS_2_ presents several polymorphs, depending on the interlayer stacking arrangement and intralayer coordination between the central Mo atom and the surrounding S atoms, the crystal structures of which are shown in Figure 1 [41,44,45]. The MoS_2_ polymorphs are identified using an adapted version of Ramsdell’s notation that was introduced for SiC polytypes [46]. This nomenclature indicates the number of S-Mo-S layers in the unit cell (1, 2, or 3) and the crystal system of the lattice, where “T”, “H”, and “R” stand for trigonal, hexagonal, and rhombohedral, respectively [47]. Five polymorphic forms have been identified for MoS_2_: 1T, 1T’, 1H, 2H, and 3R [4,33,44,48,49,50,51,52,53,54,55]. The forms 1T, 2H, and 3R are polytypes because they differ in terms of stacking configuration. The polytypes 2H and 3R occur naturally, while 1T is a metastable structure. The semiconducting 2H phase is naturally more stable and has been extensively investigated for energy-related applications [56,57], transistors [58], photodetectors [59], valleytronics, spintronic devices, and optoelectric devices [58,60,61]. In the 2H-MoS_2_ phase, each Mo coordinates with S atoms in a trigonal prismatic arrangement, with two layers in each unit cell in hexagonal symmetry, as shown in Figure 1. The semiconducting behavior of 2H-MoS_2_ results from a finite band gap between filled *d*_z_^2^ and empty *d*_x_^2^_−y_^2^_,xy_ bands [62]. The 2H-MoS_2_ phase can easily be turned into the 1T phase by exfoliating the 2H-MoS_2_ nanosheets, using the sonication-assisted lithium intercalation method, and treating the resulting material with an infrared laser [63].

With the same prismatic coordination as the 2H MoS_2_, but with three stacking layers of S–Mo–S directed along the c-axis instead of two (Figure 1), the rhombohedral 3R phase is also a naturally occurring semiconducting phase. Due to the broken inversion symmetry, this phase has drawn extensive attention because of its potential applications for non-linear optical devices [64,65,66]. Compared to monolayers, the 3R phase presents a higher predicted piezoelectricity coefficient [64]. The 3R-phase can be synthesized via homoepitaxial growth through chemical vapor deposition [67,68]. Through heating, the 3R phase can easily turn into the 2H phase [69].

The monolayered 1T, 1H and 1T’-MoS_2_ phases are other polytypes that have attracted attention but do not occur naturally. Depending on the S-atom’s geometry coordination, the Mo atom can be coordinated by six S atoms in either an octahedral (1T) or trigonal prismatic (1H) arrangement [44]. The 1T-phase can be obtained via exfoliation of the 2H-phase [70]. It is metallic in nature, with an electrical conductivity 10^7^ times higher than the 2H phase, and is of interest as a supercapacitor electrode material [70,71]. This metastable and paramagnetic phase has been reported to be efficient as a photocatalyst for hydrogen evolution and gas molecule adsorption, due to the high surface activity at the basal sites [44,45,52,53,62,70]. It is also known that the 1T phase can turn into the 2H phase with high-temperature treatment [62]. The semimetallic 1T’-phase, the more stable SLMoS_2_ with a distorted structure, due to the clusterization of Mo atoms, is formed through the dimerization of Mo atoms in the 1T phase [54]. The 1H phase is a semiconductor. It is possible to turn 1H-MoS_2_ into 1T-MoS_2_ by applying the Li intercalation method, in which an electron is transferred from the alkali metal to the *d* orbital of the transition metal center, resulting in the metallic-like character of the material [54].

The phase composition influences the photocatalytic performance of MoS_2_ nanosheets. Materials comprising 1T/2H phases have shown higher performance in the photodegradation of methyl orange dye, compared to the 2H and 3R phases [69]. Computational studies have indicated that this enhanced photocatalytic activity is due to the emergence of mid-gap states upon the introduction of 1T sites to the 2H lattice. Density functional theory (DFT)-based calculations investigating the material’s structural and electronic properties found a zero tunnel-barrier height in stable 2H/1T heterostructures, which is advantageous for achieving an efficient carrier injection rate [72].

Regarding the adsorptive properties, according to the principle of a “hard and soft acid and basis” (HSAB), soft acids (such as heavy metal ions) are capable of strong interaction with soft bases, such as sulfur atoms [73]. Thus, the MoS_2_ nanosheets show high adsorption capacity due to the abundance of adsorption sites and the fast kinetics caused by easy access to those sites [74]. Concerning solubility, MoS_2_ is soluble in aqua regia and in hot concentrated sulfuric acid, but it shows insolubility in water and diluted acid, indicating long-term stability in water systems [75].

## 3. Common Synthesis Methods and Modifications of MoS_2_

**Figure 2:**
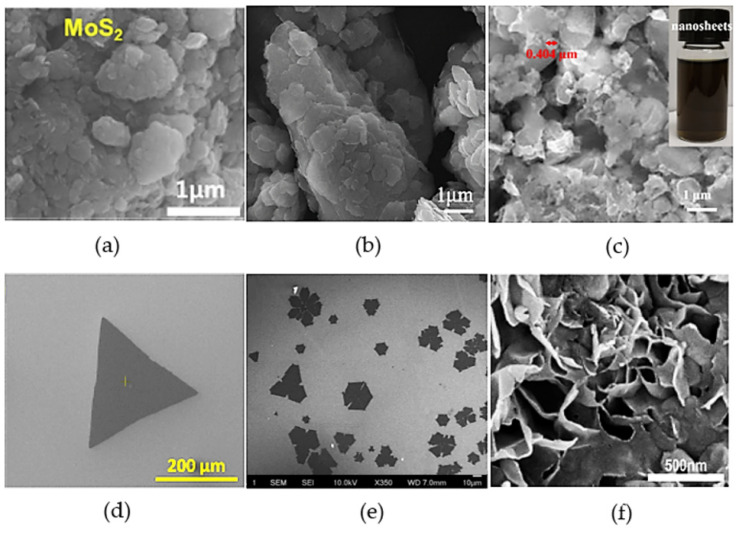
Electron microscopy images of MoS_2_, synthesized using different methods: (**a**) Ball-milling method. Reprinted from [78]. Copyright (2018), with permission from Elsevier. (**b**) Ball-milling method. Reprinted from [79]. Copyright (2020), with permission from Elsevier. (**c**) Liquid phase exfoliation method. Reprinted from [80]. Copyright (2019), with permission from Elsevier. (**d**) Chemical vapor deposition method. Reprinted from [81]. Copyright (2021), with permission from Elsevier. (**e**) Chemical vapor deposition method. Adapted from [82]. Copyright (2020), with permission from Elsevier. (**f**) Hydrothermal method. Reprinted from [83]. Copyright (2019), with permission from Elsevier.

MoS_2_ nanosheets can be synthesized through top-down methods, which consist of exfoliating the bulk MoS_2,_ and the bottom-up approach, consisting of growing nanosheets from single atoms using chemical methods such as chemical vapor deposition (CVD) or the hydrothermal method. Exfoliation processes are highly efficient and conveniently scalable, producing by-products that are less hazardous and lower costs than bottom-up methods [76,77]. However, bottom-up methods provide better control of phase composition and morphology. Figure 2 shows electron microscopy images illustrating the morphology of MoS_2_ obtained by different methods where the difference between top-down methods and bottom-up methods is visible. While the bottom-up methods present defined nanosheets (Figure 2d–f), top-down methods create structures with irregular morphology (Figure 2a–c).

### 3.1. Top-Down Methods

One of the main top-down methods is mechanical exfoliation through the ‘Scotch-tape’ method. The basis of this method is to strip the MoS_2_ flakes using tapes. The ‘Scotch-tape’ method is considered a non-destructive method in which chemical reactions are not involved [10]. Although this method has a low yield, is time-consuming, and leads to a low quantity of product, it produces SLMoS_2_ in the form of flakes and is a simple process requiring accessible equipment. This technique also enables MoS_2_ with high quality and crystallinity in 2D [10,84]. Ball-milling is another mechanical exfoliation method. This method can be employed in both dry and wet states. Dry ball-milling can be used to greatly enhance the electrochemical and catalytic properties of bulk MoS_2_ [85]. However, wet ball-milling leads to the increased production of FLMoS_2_ when compared to dry ball-milling [10]. According to Tayyebi et al. [86], the pre-functionalization of the materials can weaken the force interacting between the layers, facilitating exfoliation during the ball-milling process.

Another top-down method is liquid-phase exfoliation. In this approach, the bulk material is dispersed in a solvent and then exfoliated via mechanical processes, such as ultrasonication [87]. This method enables the high-yield creation of 2D flakes in suspension as a scalable production [88,89]. However, after long periods of inactivity or after taking the solvent out, the nanosheets could restack back into bulk form. The addition of a polymer may stabilize the MoS_2_ nanosheets, thereby maintaining the properties of exfoliated nanosheets. For instance, Deepak et al. [77] added the polymer poly(vinylidene fluoride-co-hexafluoropropylene) (PVDF-HFP) and used dimethylformamide (DMF) as a solvent in the liquid-phase exfoliation method. Due to its surface energy, water is not considered a suitable solvent for this method. However, hot water has already been employed to exfoliate MoS_2,_ with satisfactory results [87]. The liquid-phase exfoliation can be assisted by ion intercalation to enhance the yield. Ions such as lithium can penetrate the layers of MoS_2_, weakening the interlayer forces and facilitating the detachment of the layers, allowing the production of SLMoS_2_. However, this method decreases the semiconducting properties of the resulting MoS_2_ [37].

### 3.2. Bottom-Up Methods

The chemical vapor deposition (CVD) method enables a controllable route to decompose Mo and S precursors and assemble them into nanosheets [10]. The MoS_2_ nanosheets obtained by CVD are thin, of high quality and high purity, and present limited defects as 2D materials [90]. This method is also indicated for producing SLMoS_2_ in large quantities at a low cost. However, it presents low control in terms of target products [91] and lacks good reproducibility since it depends on various parameters, such as temperature, pressure, and reaction time, among others [81]. The possibility of forming both SLMoS_2_ and FLMoS_2_ zones is also a limitation.

An alternative bottom-up approach that is frequently used is the hydrothermal method. In this method, the MoS_2_ crystallizes from aqueous or organic solutions processed at high pressure and temperature [10]. This method achieves high yield, controllable size, and uniform thickness [10]. Although the hydrothermal method is widely used, the mechanism is unclear as the reaction occurs in a closed environment, an autoclave [10]. Different precursors can be used in this method. Khabiri et al. [92] used the hydrothermal method to prepare composites containing MoS_2_ nanoflower structures, consisting of adding ammonium molybdate, thiourea, and water to an autoclave and setting the temperature to 180 °C for 20 h. Other authors used a similar procedure, but added ammonium tetrathiomolybdate, ethanol, and water to an autoclave heated at 200 °C for 10 min [93].

**Figure 3:**
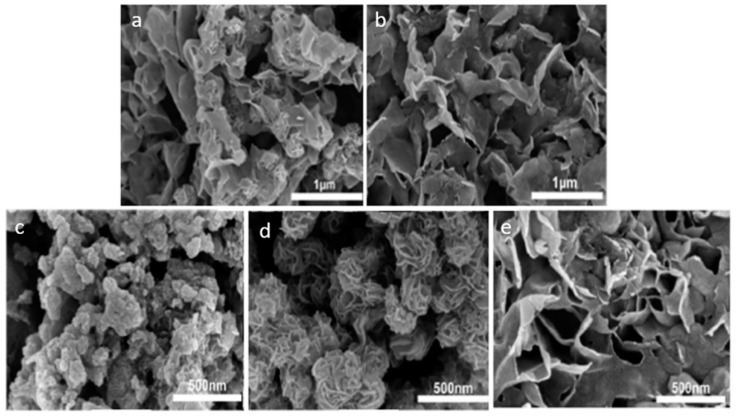
SEM images of MoS_2_ composites synthesized via the hydrothermal method. (**a**) S/Mo molar ratio 1:1; (**b**) S/Mo molar ratio 4:1; (**c**) synthesis time: 25 h at 150 °C; (**d**) synthesis time: 37 h at 180 °C; (**e**) synthesis time: 47 h at 240 °C. Reprinted from [83]. Copyright (2019), with permission from Elsevier.

In the hydrothermal method, the morphology and properties of MoS_2_ can be modified by adjusting the experimental synthesis conditions. Luo et al. [83] observed different morphologies, depending on the S:Mo molar ratio of the starting materials of molybdenum oxide (MoO_3_) and potassium thiocyanate (KSCN). Low ratios led to the formation of nano-flower structures (Figure 3a), while at higher ratios (Figure 3b), only nanosheet structures were observed. The temperature and time of the synthesis can also influence the morphology. It was found that at a lower temperature (150 °C) for 25 h, the resulting morphology was like a coral (Figure 3c); when increasing either the time or temperature, the dominant morphology was a flower-like structure (Figure 3d), with size increasing over the synthesis time. At higher temperatures and longer reaction times (240 °C for 47 h), the observed structure consisted of large nanosheets, showing the largest surface area. Other structures, such as core-shell or hollow structures, have been produced, aiming to enhance the properties of MoS_2_ [94,95]. Wu et al. [95] produced 2-µm diameter hollow MoS_2_ microspheres via the hydrothermal method and used this material to adsorb the organic contaminant, methyl orange.

### 3.3. MoS_2_ Modification Methods

The properties of MoS_2_ can be modified by expanding the interlaying spacing, a strategy that has been applied in the fields of batteries and photocatalysis [96]. Ai et al. [97] produced a widened defect-rich nano MoS_2_ (W-DR-N-MoS_2_) via the hydrothermal method to adsorb mercury (Hg^2+^). The interlayer spacing could be controlled by the synthesis temperatures. The produced MoS_2_ consisted of layers of MoS_2_ with a wider space between them (d = 9.4 Å against d = 6.15 Å for MoS_2_ powder); empty spaces in the layers also characterized this material. Both aspects improved the adsorption capability by enabling adsorption between the layers.

The combination of MoS_2_ with other components can lead to materials with improved adsorption capacity. For example, kaolin was used as a substrate for MoS_2_ growth via the hydrothermal method. The resulting composite showed increased pore volume, a larger number of adsorption sites, and a consequently greater Pb(II) adsorption efficiency than their counterparts [98]. On the other hand, using montmorillonite (MMT) decreased the hydrophobicity of MoS_2_, increasing the dispersibility in water and improving the adsorptive removal of Hg(II) [99]. The growth of Fe_3_O_4_ nanoparticles onto MoS_2_ nanosheets resulted in MoS_2_ composites with enhanced conductivity and magnetic behavior [100,101].

Doping MoS_2_ with heteroatoms can also modify the MoS_2_ properties by regulating the electronic structure and conductivity, regulating the interlaying spacing, and increasing the number of active sites [102,103,104]. This type of modification has been shown to be effective in photocatalytic hydrogen production and battery fields [102,103,104]. Doping with metallic elements may lead to undesirable consequences, such as the reduction in stability of MoS_2_ by promoting the formation of the MoS_3_ phase. In contrast, using non-metallic elements promotes an increased number of active sites and conductivity [103]. Xin et al. [102] synthesized P-doped MoS_2_ for photocatalytic hydrogen production. P-doped MoS_2_ exhibited broader spectra absorption and a hydrogen production rate 2.8 times higher than the MoS_2_ counterpart.

Phase engineering is an approach in which several phases are combined to obtain optimal MoS_2_ properties. As mentioned above, a combined 1T/2H phase achieved better results in the photodegradation of methyl orange due to the increased conductivity of the 1T phase and the defects present in this phase [69]. An identical MoS_2_ phase combination was used in the photodegradation of potassium hexyl dithiocarbonate, which is an organic pollutant in mine wastewater [105].

An alternative strategy to modify the MoS_2_ properties is defect engineering, which consists of creating defect sites in MoS_2_ layers to act as active sites for catalysis [69,97]. Luo et al. [106] produced MoS_2_ with S defects using a ball-milling method in ascorbic acid, an organic acid-reducing agent. The resulting S defect-rich ultrathin MoS_2_ nanosheets were used to photo-reduce Cr(VI). This procedure leads to the formation of two types of S-defects: point defects that may act as recombination centers for photogenerated carriers, and stripping defects that promote the separation of photogenerated electron-hole pairs. According to the authors, combining both types of defects led to an enhancement of photocatalytic performance.

In the normal MoS_2_, only the edges sites have active regions with catalytic activity. One way to enhance this property is via the construction of vertically oriented MoS_2_ nanosheets (V-MoS_2_), which exhibit higher amounts of edge sites, enhanced longitudinal in-plane carrier transport, and stronger light adsorption [107,108]. Liu et al. [107] produced V-MoS_2_ by means of CVD induced by a TiO_2_ buffer layer, for application in photodetectors. Cui et al. [108] successfully applied a vertical growth MoS_2_ construction method in graphene sheets to increase the electrochemical performance of lithium-sulfur batteries.

## 4. Adsorption Applications

This section reviews the application of MoS_2_-based nanomaterials to clean up emerging pollutants from water using adsorptive technologies. Table 1 provides an overview of several MoS_2_-based nanomaterials investigated for the adsorption of inorganic and organic contaminants.

### 4.1. Heavy Metal Species

Pollution with heavy metal species is a huge concern because it represents a high risk for the environment and for humans [8]. Heavy metal ions are toxic, non-biodegradable, persistent in the environment, and tend to accumulate in organisms, causing health issues [8]. Lead (Pb(II)) is one of the most common ions found in industrial wastewater and shows toxicity, even at low concentrations (≥15 µg/L in drinking water) [8]. Many problems, such as anemia, hypertension, reduced intelligence quotient, and immunotoxicity are related to Pb(II) exposure [8]. Mercury is another heavy metal that presents a high risk to human health and may cause kidney failure, severe pulmonary irritation, neurological disorders, and death [73]. Mercury exists in the form of several inorganic (e.g., metallic mercury or mercuric salts (Hg(II)) and organometallic species (e.g., methylmercury). The Hg(II) species can also affect the food chain once they can bioaccumulate [73]. Chromium, particularly chromium (VI), is hazardous to human health as well [112]. In this oxidation state, Cr(VI) can cause cancer and fetal malformations with only a concentration higher than 0.10 mg/L in drinking water [112].

As stated above, MoS_2_ has good adsorption capability for heavy metal ions due to the sulfur atoms on the surface. For this reason, MoS_2_ has been investigated in terms of the adsorptive removal of these pollutants from water. Table 1 lists the most relevant studies. Most of the studies focused firstly on Hg(II) removal, due to its high toxicity, and, secondly, on Pb(II) and Cr(VI) removal. For instance, Pirarath et al. [73] produced nanosheets of MoS_2_ via surfactant-assisted hydrothermal synthesis, using sodium dodecyl sulfate (SDS), and investigated the adsorption of Hg(II) in batch conditions. The negative charge of the S layers increased with SDS, which induced an electrostatic interaction between Hg(II) and MoS_2_. A higher adsorption capacity was achieved due to the excess negative charge of the S layer, compared to the nanosheets of MoS_2_ produced by other methods. Within 300 min, 0.5 g/L of the composite enabled 93% removal of Hg(II) in distilled water samples. Ai et al. [97] produced a widened defect-rich nano MoS_2_ (W-DR-N- MoS_2_), with many sulfur binding sites exposed due to enlarged interlayer spacing. With this modification, Hg(II) removal of 99.8% was achieved in just 5 min in distilled water samples, using 0.1 g/L of the material. The Hg(II) removal was also efficient in both natural water and industrial wastewater samples. The recovery of the adsorbent is economically important. Coupling a magnetic component to MoS_2_ has been investigated as a strategy to facilitate its separation; employing a magnetic field is an accessible method. Zhi et al. [109] produced an aerogel of MoS_2_ and GO with embedded Fe_3_O_4_ and Au NPs (Au/Fe_3_O_4_/MoS_2_CAs) to remove Hg(II). A removal performance of 100% was achieved in only 30 min with 0.5 g/L of Au/Fe_3_O_4_/MoS_2_CAs, which is a great achievement for Hg(II) adsorption. The reuse of this composite for up to 10 cycles only slightly decreased the efficiency of the adsorption. Wang et al. [111] used flower-like MoS_2_ decorated with Fe_3_O_4_ to investigate the simultaneous removal of Hg(II) and Pb(II) from distilled water, along with Pb(II) removal in real battery wastewater and soil samples, achieving satisfactory results. Yuan et al. [98] produced a MoS_2_-kaolin composite for Pb(II) adsorption, reaching a 99.9% removal rate in 40 min with 1.6 g/L of composite. According to the authors, using kaolin induces uniform MoS_2_ growth and a higher pore volume, leading to more adsorption sites. Regarding the Cr(VI) removal, Chen et al. [112] produced MoS_2_/lignin-derived carbon (MoS_2_/LDC) and achieved 99.4% removal of Cr(VI) in distilled water samples after 30 min, using just 0.10 g/L of composite.

It is essential to evaluate the selectivity of the adsorbents toward the target pollutant because in natural water or wastewater, there are other species that can compete for sorption sites. In this context, Zhi et al. [109] evaluated the selectivity of the magnetic Au/Fe_3_O_4_/MoS_2_CAs composites toward Hg(II) capture via a mixture of a large number of ions in a synthetic water sample. Despite the presence of several ions (Na(I), K(I), Ag(I), Ca(II), Ba(II), Mg(II), Fe(II), Cu(II), Zn(II), Ni(II), Cd(II), Al(III), Pb(II), Cr(III) and Hg(II)) the most adsorbed ion was Hg(II). All the other ions did not show relevant adsorption. The reason for this selectivity may be related to the HSAB principle, i.e., soft acids such as Hg(II) establish strong interactions with soft bases such as sulfur [111,115].

After adsorption tests, the composite with the adsorbed heavy metal ion can be separated from the solution and treated, aiming for a reduction in the heavy metal ions, which precipitate and desorb from the catalyst [111,112].

Besides the heavy metal species, MoS_2_-based materials have been employed for the capture of noble metal species, namely, silver and gold ions. The recovery of these metals from industrial effluents and wastewater is important, not only to prevent pollution but also because of their economic value [28,74]. Zeng et al. [74] produced a 2D MoS_2_ to remove Ag(I) from distilled water samples. The mechanism of adsorption was investigated using X-ray photoelectron spectroscopy (XPS) and DFT calculations, confirming a strong interaction between MoS_2_ and Ag(I) by the complexation of Ag-S and Ag-O through hybridization between the d orbital of Ag and the p orbital of S or O [74]. The recovery of gold ions (Au(III)) has also been investigated [28]. Using chitosan-coated MoS_2_ (0.28 g/L dose), Zhao et al. [28] achieved 98.9% ion removal in 300 min. The ion-selectivity of chitosan-coated MoS_2_ in wastewater containing Au(III), Cu(II), Mg(II), Ni(I), Li(I), and Zn(II) species was evaluated. It was found that the biosorbent presented the highest selectivity to the Au(III) ions and the lowest affinity to Zn(II) ions. After adsorption, desorption enabled the recovery of noble metal ions for further use.

### 4.2. Organic Contaminants

The adsorption of organic contaminants using MoS_2_-based materials was less widely investigated. MoS_2_-based membranes for organic contaminant removal are beyond the scope of this work and have recently been reviewed elsewhere [13]. Most research papers that present results of the adsorption of organic contaminants have, as their primary focus, the study of the photodegradation of these contaminants. Table 1 lists several studies of the adsorption of organic contaminants. MoS_2_ nanosheets produced by the hydrothermal method removed nearly 82.3% of Bisphenol A (BA), which is an endocrine disruptor, after 120 min with a sorbent dosage of 1.5 g/L [83]. Reduced graphene oxide-MoS_2_ (rGO-MoS_2_) composites successfully removed the ofloxacin antibiotic [7]. Despite the low surface area (17 m^2^/g), 95% removal was achieved after 240 min with 0.35 g/L of adsorbent. The adsorption capacity in real wastewater samples was also investigated. However, the removal drastically decreased after composite reuse, and only 4% was removed after 10 cycles. Gao et al. [113] produced a composite with a metal–organic framework (UiO-66/MoS_2_) and report a comprehensive study on the adsorption and photocatalytic degradation of lomefloxacin. The beta-blockers, atenolol and acebutolol, were also adsorbed successfully in distilled water with MoS_2_/montmorillonite (MoS_2_/MTT) composites [114]. Regarding the adsorption of dyes, methyl orange was adsorbed by hollow MoS_2_ microspheres (h-MoS_2_) [95]. The adsorption equilibrium was achieved in just 10 s for MO, with a maximum adsorption capacity of 42 mg/g.

MoS_2_-based composites have also been used for the adsorption of various organic dyes. Song et al. [100] produced MoS_2_ decorated with Fe_3_O_4_ nanoparticles and tested this composite for the adsorption of Congo red (CR), methylene blue (MB), methylene green (MG), rhodamine B (RhB) and eosin Y (EY). Among these dyes, the composite showed higher adsorption of CR with an adsorption capacity of 71 mg/g, achieving the removal of about 65.2% in 2 min, which increased to 71% after 120 min with 1 g/L of the composite.

### 4.3. Kinetic and Isotherm Studies

Table 1 presents a summary of relevant information on the kinetics and equilibrium adsorption tests employing MoS_2_-based materials. Overall, the kinetic model that better described the results was the pseudo-second-order (PSO) kinetic model, with an R^2^ value of between 0.901–1.000, indicating a good fit of this model to the adsorption data of both the metal species and organic contaminants. Regarding the equilibrium description, the Langmuir model better described the adsorption data (R^2^ = 0.86–1.0). However, the equilibrium adsorption of the antibiotic lomefloxacin and MO dye was better described by the Freundlich isotherm model, indicating non-monolayer formation [83,115]. Wang et al. [111] used flower-like MoS_2_ decorated with Fe_3_O_4_ nanoparticles in the removal of Hg(II) and Pb(II) from an aqueous environment. The maximum adsorption capacity estimated by the Langmuir model was 428.9 mg/g for Hg(II) and 263.6 mg/g for Pb(II), under optimized conditions (pH 5, 25 °C, 0.8 g L^−1^ sorbent dosage). The adsorption kinetics were well described by the PSO model and the good fitting of the Langmuir isotherm model was in line with monolayer formation. The favorable adsorption capacity, selectivity, and recyclability originated from the strong interaction between S and the heavy metal ions. The MoS_2_-clinoptilolite composite removed nearly 100% (99.8%) of Pb(II) from aqueous solution (pH 6, 25 °C, starting concentration of 50 mg L^−1^, 1.5 g L^−1^ sorbent dosage) after 90 min [8]. This adsorption was ascribed to the interaction of Pb(II), not only with the S layers of the MoS_2_ but also with the -OH and -COOH functional groups of clinoptilodite. The thermodynamic parameters were calculated and indicated that the adsorption was spontaneous and exothermic in nature.

Looking at the equilibrium adsorption values (*qe*) of the metal species in Table 1, the highest value found was 3435 mg/g (R^2^ = 0.991) for the adsorption of Au(III) at an optimal pH of 5, using chitosan-coated MoS_2_ (CS/MoS_2_) crosslinked with glutaraldehyde. The value was estimated by the Langmuir model and was close to the experimental value (3109 mg/g) [28]. The adsorption capacity increased and the adsorption isotherm changed from multilayer to monolayer when the content of MoS_2_ increased. The highest adsorption capacity was observed for a mass ratio of 1:2 (CS:MoS_2_), at 35 °C, after 300 min. The high efficiency of these materials to adsorb Au(III) was ascribed to electrostatic interaction, in the form of tetrachloroaurate ions (AuCl_4_^-^) and then to complexation by the sulfur- and nitrogen-containing functional group. The materials also showed outstanding selectivity for gold ions in the presence of coexisting ions and demonstrated attractive reusability after four cycles.

Concerning the organic contaminants, the highest adsorption capacity was observed for atenolol, using MoS_2_/MTT as an adsorbent (*qe* = 146 mg/g) and close to the value estimated by the Langmuir model (132 mg/g) [114]. The adsorption kinetics could be described using the PSO model. The interactions leading to the adsorption included van der Waals interactions and hydrogen bonding between the hydroxyl groups of the atenolol and the sorbent. The quantum chemical calculations results were in line with the lower adsorption capacity observed for acetobutolol using the same adsorbent.

Other parameters and isotherm models have been used to characterize the adsorption, such as the distribution coefficient, which expresses the affinity of a compound to the adsorbent, and Temkin isotherm, which assumes that the heat of adsorption does not remain constant during the adsorption process [74,110].

## 5. Photocatalytic Applications

Adsorption is an excellent approach to removing pollutants from wastewater. However, this technique does not provide a complete solution to the problem since the contaminants are not mineralized. In this sense, advanced oxidation processes (AOPs), such as photocatalytic oxidation, have been developed to promote the degradation of pollutants [116].

Due to the relatively small band gap of MoS_2_, visible-light irradiation can induce a catalytic chain reaction. For bulk MoS_2_, the band gap is ~1.2 eV, which means that MoS_2_ is activated with almost all the solar spectrum, while SLMoS_2_ shows a band gap of ~1.8 eV, this phase being activated by a radiation wavelength of <660 nm. However, as the bulk MoS_2_ has unsaturated Mo and S atoms at the edge, leading to an indirect and small band gap, this is insufficient to achieve photocatalytic reactions. For this reason, bulk MoS_2_ is not suitable for photocatalysis [117].

**Figure 4:**
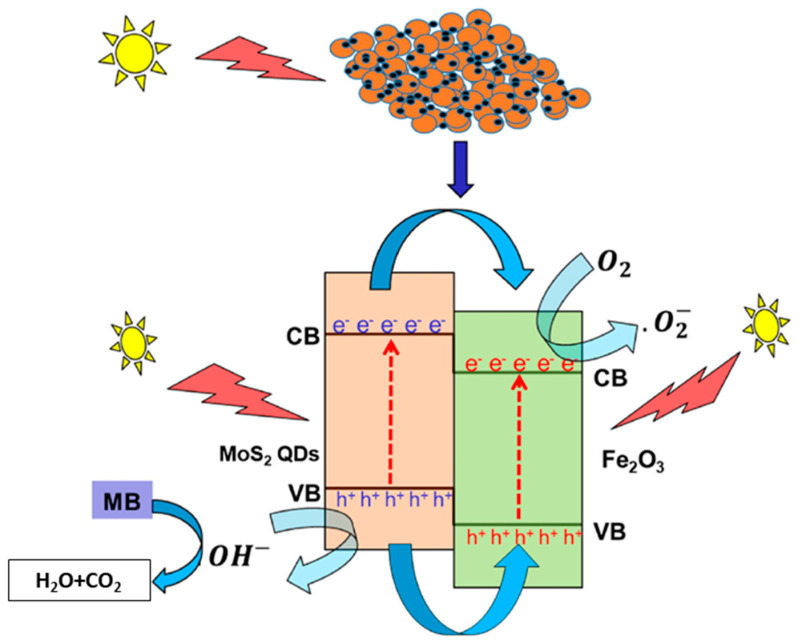
Schematics of the proposed photocatalysis mechanism of α-Fe_2_O_3_/MoS_2_QDs. Reproduced from [92], with permission from John Wiley & Sons. Copyright 2018 Wiley-VCH GmbH.

MoS_2_ has been combined with several components to improve its performance as a photocatalyst to degrade contaminants. Table 2 lists several recent studies on the photocatalytic degradation of pollutants in water. MoS_2_-based materials have been primarily employed in the photodegradation of dyes. Dye-related industries are reported as the major source of contaminated wastewater [118]. Dye-containing wastewater presents both chromaticity and toxicity [25,119]. Pharmaceuticals, antibiotics, and pesticides, among other chemical compounds, also represent emerging contaminants where photodegradation has been investigated, due to their toxicity and increasing detection in wastewater [119]. The same photocatalyst could photodegrade various contaminants at different rates. For example, Fu et al. [120] reported degradation rates above 70% for methylene blue (MB), methyl orange (MO), and Congo red (CR), using ZnO/MoS_2_ nanoarrays. In contrast, the degradation of rhodamine B (RhB) dye was only around 8%. Regarding the degradation of MB, Khabiri et al. [92] achieved 83% degradation after 1 min of irradiation with VIS light, that increased to 91% after 120 min using α-Fe_2_O_3_/MoS_2_ QDs, a composite comprising hematite (α-Fe_2_O_3_) and nanosized MoS_2_ (MoS_2_ quantum dots (QDs)). Figure 4 illustrates the proposed photocatalytic mechanism. The visible light irradiation excites the electrons (e^−^) in the valence bands (VB) of α-Fe_2_O_3_ and MoS_2_QDs to the respective conduction bands (CB), leaving a hole (h^+^) in the VB. The CB and VB positions of MoS_2_QDs are above α-Fe_2_O_3_. Due to the potential energy difference, the photogenerated electrons at the CB of MoS_2_QDs transfer to the CB of α-Fe_2_O_3_, then migrate to the surface of the catalyst, which enables the creation of the reactive species as a superoxide anion radical (·O_2_^−^) by the reaction with O_2_, and hydroxyl radicals (·OH) from reaction with water. The reactive species react with MB, thereby degrading it [92]. Most of the values for the degradation rate presented in Table 2 are above 70% for different contaminants, MoS_2_-based catalysts, and methodologies. For MB, the highest degradation rate was 97%, after 40 min of reaction using 1 g/L of MZO (MoS_2_/ZnO) [121]. A similar degradation rate was obtained in just 5 min using the ternary catalyst MoS_2_/BiFeO_3_/Ag_3_PO_4_ under ultraviolet-visible (UV-Vis) irradiation [119]. The same catalyst achieved a good degradation of MO and RhB, with 98% and 97% degradation, respectively, after 5 min of irradiation (Table 2) [119]. Regarding other contaminants from the antibiotic spectrum, tetracycline (20 mg/L) was successfully degraded up to 99% within 50 min under visible light, catalyzed with 0.4 g/L MoS_2_/Eu/B-g-C_3_N_4_ (MoS_2_/BEuCN) as the catalyst [122]. Ahamad et al. [123] studied the photocatalytic degradation of bisphenol-A (BA) using the composite g-C_3_N_4_/MoS_2_−PANI, achieving 93% of removal after 60 min under visible light. DFT calculations were performed to elucidate the photocatalytic degradation mechanism of BA. It was proposed that the aromatic ring would be one of the most likely sites for an attack by photo-generated radicals, which leads to several intermediate species and to the production of the final products, CO_2_ and H_2_O [123].

Although most photodegradation studies rely on organic contaminants, MoS_2_-based photocatalysts were also employed to reduce Cr(VI) to Cr(III) [120,134]. As mentioned before, Cr(VI) is a highly toxic form of chromium. By reducing it to Cr(III), the toxicity decreases, and the chromium can be precipitated and removed [144]. Via photo-reduction, 85% Cr(VI) removal was achieved using MoS_2_/BiOBr/CF under visible light after 2 h, and 75% Cr removal rate using Ni foam with ZnO/MoS_2_ after 25 min under UV-VIS light [120,134]. Shi et al. [134] tested the effect of pH in this photocatalytic reaction and observed the highest removal (91%) at pH = 3 and the lowest removal (29%) at pH = 11. This behavior was ascribed to different chromium speciation according to pH. At pH < 7, the predominant species is the dichromate anion (Cr_2_O_7_^2^^−^), which is more likely to be reduced to Cr(III) than the chromate anion (CrO_4_^2−^), which predominates at pH > 7. In addition, using MoS_2_/TiO_2_/graphene, Chen et al. [137] achieved an 88% removal of Pb(II) in an aqueous solution in just 60 min under UV-Vis light. The removal was higher in an acidic environment (pH < 7) because the dominant species at those pH values (Pb^2+^(H_2_O)_6_) is more reactive than the other Pb(II) species, due to the more positive charge and smaller hydration radius.

In photocatalysis, the goal is to achieve the total mineralization of the pollutants. This means fully converting the contaminants to a gaseous form, water, salts, and minerals [145]. However, several photodegradation reactions may lead to toxic intermediate compounds, which can harm the environment [146]. The total organic carbon (TOC) quantification method can be employed to evaluate the mineralization of a contaminant achieved by photocatalysis. Table 3 shows a relatively high mineralization rate, with more than 54% of the mineralization provided by MoS_2_-based photocatalysts. This means that most of the contaminant molecules present in the solution (>50%) were fully degraded, while part may still be the original contaminant (when the removal rate is less than 100%) or is converted into some intermediate compound. For example, 100% degradation of Rhodamine B was achieved with the BiOI/MoS_2_ (BMS-8) catalyst. However, only 78% of the initial amount of the dye was fully mineralized, leaving 23% in the form of intermediate compounds [3]. Overall, the results show the promising potential of MoS_2_-based catalysts to photodegrade and mineralize the organic contaminants in wastewater systems.

## 6. MoS_2_-Based Nanocomposites

Although nano-MoS_2_ shows good efficiency as a photocatalyst for removing several contaminants, it can be enhanced when in combination with other components in the form of nanocomposites. Table 4 lists several works comparing the photodegradation provided by bare nano-MoS_2_ and MoS_2_-based nanocomposites. Overall, MoS_2_-based nanocomposites present a higher specific area when compared to bare nano-MoS_2_. The increase in the specific surface area of the catalyst can contribute to an enhanced removal rate once a higher exposed specific area favors the contaminant-catalyst contact [142].

Overall MoS_2_-based composites presented better adsorptive performance than bare MoS_2_. The creation of defects and increasing interlayer spacing were used to enhance the adsorption capacity of Hg(II) from 36 mg/g to 2563 mg/g [97]. The introduction of montmorillonite (MMT) enhanced the water dispersibility of MoS_2_ by decreasing the hydrophobicity, which improved the adsorption capacity [99]. For instance, MoS_2_/MMT composites adsorbed nearly 1120 mg/g of Hg(II), which is markedly higher than the value observed for bare MoS_2_ (937 mg/g). MoS_2_/MMT composite was also used to adsorb the beta-blockers atenolol and acebutolol, achieving good results [114]. Kaolin has been used as a substrate, providing a uniform growth of MoS_2_ and well-distributed active sites, which improved the removal of Pb(II) from 55% in bare MoS_2_ to 89% in MoS_2_-kaolin [98].

The combination of MoS_2_ with semiconducting phases has been explored, to improve photocatalytic activity. Quan et al. [121] coupled MoS_2_ nanoflowers to ZnO nanoparticles and observed a considerable increase in the degradation rate of methylene blue compared with bare MoS_2_, from 44% to 97%, when submitted to visible light (λ > 420 nm). Introducing the semiconductor ZnO resulted in an increased photoinduced electron transfer rate, leading to higher photocatalytic activity. ZnO also presents attractive characteristics, such as low cost, low toxicity, high chemical stability, and strong photosensitivity [121]. Mahalakshmi et al. [127] produced core@shell TiO_2_@MoS_2_ heterojunction composites via the one-step hydrothermal method. The photocatalytic activity of the resulting composite toward 4-nitrophenol was enhanced compared to the MoS_2_ and TiO_2_ used separately, due to a reduced band gap and efficient separation of the photogenerated electron-hole pairs. A similar strategy was reported for coupling MoS_2_ and CuO, with improved separation of electron-hole pairs [142]. A Z-scheme heterojunction was produced that promoted the separation of the photogenerated carriers and may also increase the specific area and, consequently, the number of active sites [142]. BiOI nanoplates were also combined with MoS_2_ nanosheets [3]. ZnS was combined with MoS_2_ to increase the efficiency of photocarrier generation, increasing the removal of crystal violet dye using visible light (λ > 420 nm) [132]. Composites of MoS_2_ with UiO-66 resulted in an enhanced specific area and in more active sites being available [113]. Several studies report the incorporation of magnetic nanostructures (e.g., magnetite—Fe_3_O_4_) to enhance the photocatalytic activity of MoS_2_ [25,135]. This approach facilitates the separation of the nanocatalyst from the treated solution and increases the efficiency of the transport of photogenerated electrons, leading to a higher removal rate, even in the case of a lower specific area compared to bare MoS_2_ [25].

## 7. Reuse of MoS_2_-Based Composites for Adsorption and Photocatalysis

After interacting with the contaminated solution, the catalysts and adsorbents should then be collected and reused to reduce the quantity of produced materials and the associated costs. Importantly, the composite should maintain good adsorption/photoactivity when reused. Reusability studies have been performed to verify the efficiency of the composite after several cycles. At the end of each removal experiment, the sorbent/photocatalyst is collected through centrifugation or filtration and is washed off with water and ethanol or water alone, and then dried [121,124]. In this context, magnetic nanomaterials are advantageous because they are easily recovered using magnetic separation, which requires less energy [25,100,111,135].

Table 5 compares the evolution of the degradation rate via adsorption and photodegradation after several testing cycles. The MoS_2_-based composites showed good adsorption when comparing the first and the last cycles [99,110,111,112]. The Au/MoS_2_ composites also presented good adsorption after four consecutive cycles in a solution containing multiple ions, demonstrating their great potential for heavy metal ion removal [28]. Zhi et al. [109] reported a removal rate of > 95% after 10 reusing cycles for the adsorption of Hg(II). Several other studies report minor differences in adsorption capacity at the first and last adsorption cycles, regardless of the contaminant. Conversely, a marked decrease in the adsorption of the antibiotic ofloxacin was observed when the rGO-MoS_2_ composite was reused [7]. The removal decreased from 92% to just 4% after 8 cycles, but the authors do not offer a possible explanation [7].

The reuse of MoS_2_-based photocatalysts has also been investigated. However, often, the papers do not give detailed results of the reuse studies. Overall, the photocatalyst maintains good photocatalytic activity after several cycles. Yet, in some cases, significant decreases in the photodegradation rate were observed due to the oxidation of the photocatalyst [126].

## 8. Application of MoS_2_-Based Composites in Real Environmental Samples

So far, most studies have only focused on treating singular contaminant solutions in synthetic aqueous samples. The number of reports testing MoS_2_ and composites in water decontamination in more realistic conditions is still scarce. MoS_2_/Fe_3_O_4_ composite was employed as an adsorbent to treat effluents from lead-acid battery factories, reaching a Pb(II) removal of 99.6% and showing acceptable Pb(II) removal in contaminated soil [111]. Regarding the adsorption of organic contaminants, Zeng et al. [147] used MoS_2_-decorated biochar to remove the antibiotic tetracycline hydrochloride from river and tap water. The adsorption capacity was found to be higher in environmental water samples when compared to synthetic solutions, prepared in deionized water and used under the same conditions. Furthermore, the composite could be reused for 5 cycles with only a minor decrease in the adsorption capacity. Conversely, the adsorption capacity of the antibiotic ofloxacin by the rGO-MoS_2_ composites in river water was lower than in deionized water [7]. The effect was ascribed to the presence of cations, natural and synthetic organic chemicals that compete with ofloxacin for adsorption, leading to a decrease in the adsorption capacity. Huang et al. [148] realized that the photocatalytic activity of MoS_2_ microspheres in degrading thiobencarb in lake or river water decreased by 20% compared to its performance in spiked deionized water, under identical reaction conditions. The presence of anions in non-deionized water acting as scavengers was identified as a cause for the decrease in photocatalytic activity. Nevertheless, the authors considered this material a potential catalyst for removing pollutants. Chandrabose et al. [149] assessed the photocatalytic efficiency of MoS_2_/TiO_2_ composite in a solution comprising a mixture of anionic dyes (rhodamine B and methyl orange) and cationic dyes (methylene blue and crystal violet). The composite removed 100% of cationic dyes and 70% of the anionic dyes in the first stage of adsorption in the dark, while the remaining 30% of anionic dyes were photodegraded entirely after 3.5 h in a second stage under UV-Vis light irradiation. The composite showed higher adsorptive affinity to the cationic dyes compared to anionic dyes. Increasing contaminant concentrations resulted in less adsorptive removal at the first stage, which was limited by the surface area of the composite. The results of the study show the potential of using this MoS_2_-based composite for the removal of dyes produced by textile industries.

## 9. Environmental Impact of MoS_2_-Based Composites

Water remediation studies employing MoS_2_-based composites have been focused on the evaluation and enhancement of the composites’ removal capacity. The assessment of the ecotoxicity of the materials has been less widely investigated, although it is a fundamental matter. Nanomaterials have emerged as a promising tool for water remediation, but, due to their high surface area and chemical activity, if accidentally released into the environment, they may constitute a risk [150]. Depending on their chemical properties and composition, nanomaterials can be modified by oxidation, sulfidation, aggregation, and deposition in the natural environment [151]. In terms of the properties of nano-MoS_2_, this material is considered chemically stable even in environmental conditions [75]. Due to its low solubility in water systems, it is also considered a persisting compound. Nano-MoS_2_ may persist in the environment and the actual risks are still unknown. The nanosheets of MoS_2_ showed antibacterial activity against *Escherichia coli* and *Staphylococcus aureus* and low cytotoxicity for Vero cells [19]. Cicuendez et al. [152] also observed good viability of mouse and human cells when exposed to MoS_2_ flakes. A more comprehensive study on the toxicity of bulk and MoS_2_ nanosheets was conducted to address the toxicity in various aquatic species of different taxonomic groups [34]. The EC50 was determined for four species: *Vibrio fischeri*, a marine Gram-negative photobacterium, *Pseudokirchnerialla subcapitata*, a freshwater microalga, *Daphnia magna*, a freshwater crustacean, and *Spirodela polyrhiza*, a freshwater duckweed. Bulk and nano MoS_2_ (0.05–2.00 g/L) were tested for a contact time ranging from 15 min to 72 h. It was found that bulk MoS_2_ was more toxic than MoS_2_ nanosheets for all the species tested; in some cases, nano MoS_2_ showed no toxicity for the tested conditions.

## 10. Conclusions and Perspectives

In summary, this review highlights the advantages of MoS_2_ and MoS_2_–based nanomaterials for water treatment applications through adsorption and photocatalysis. Owing to the high surface area and visible light-responsive photocatalytic activity, we can expect growing interest in these materials for environmental applications in the near future. Several advances have been made in synthetic methods for the production of MoS_2_ nanomaterials with different structural features, namely, the crystal phase, morphology and layer spacing, which each create distinct physicochemical properties. The applications of MoS_2_ and composites in the adsorption and photodegradation of metal ion species and organic contaminants are thoroughly summarized. The modification of MoS_2_ and its combination with other components enhance those properties that increase removal efficiency and may also facilitate the separation of the catalyst from the solution. Despite the progress achieved, more studies are needed to rationally fabricate highly efficient MoS_2_-based photocatalysts, which represents an opportunity for further research efforts. The ecotoxicity of MoS_2_ is still not clear and requires further investigation. Although MoS_2_ presents high chemical stability, even in environmental aqueous systems, it also presents some toxicity to different organisms. More studies should be conducted to assess the risks of employing MoS_2_-based materials in water treatment.

## Figures and Tables

**Table 1:** Kinetics and equilibrium parameters from the fitted models in adsorption studies for the removal of metal ion species and organic contaminants, using MoS_2_-based composites.

Composite	MoS_2_ Preparation Method	Adsorption Compound	Temperature (°C)	Adsorption Kinetics			Adsorption Isotherms				Ref.
					PFO	PSO	Langmuir		Freundlich		
**Metal species**											
MoS_2_	HT	Hg(II)	27	q_e_ (mg/g)	208.5	303					[73]
				k_1_ (min^−1^)	0.032	-					
				k_2_ (g/(mg min))	-	2.710					
				R^2^	0.941	0.998					
Au/Fe_3_O_4_/MoS_2_CAs	Commercial	Hg(II)		q_e_ (mg/g)	0.479	20.408	q_m_ (mg/g)	1527	n	3.040	[109]
				k_1_ (min^−1^)	0.089	-	K_L_ (L/mg)	0.083	K_F_ (mg/g)	200.85	
				k_2_ (g/(mg min))	-	1.2	R^2^	0.999	R^2^	0.774	
				R^2^	0.779	0.999					
3D MoS_2_-rGO	HT	Hg(II)	25	q_e_ (mg/g)		30.55	q_m_ (mg/g)	400	n	1.046	[110]
				k_1_ (min^−1^)	0.117	-	K_L_ (L/mg)	65.68	K_F_ (mg/g)	5.936	
				k_2_ (g/(mg min))	-	3.75 × 10^−3^	R^2^	0.9998	R^2^	0.999	
				R^2^	0.785	0.999					
W-DR-N-MoS_2_	HT	Hg(II)		q_e_ (mg/g)	-	-	q_m_ (mg/g)	2562.8	n		[97]
				k_1_ (min^−1^)	-	-	K_L_ (L/mg)	3.029	K_F_ (mg/g)		
				k_2_ (g/(mg min))	-	1.42	R^2^	0.999	R^2^		
				R^2^	-	0.9999					
MoS_2_/Fe_3_O_4_	HT	Hg(II)	25	q_e_ (mg/g)	84.46	254.3	q_m_ (mg/g)	428.9	N	198.8	[111]
				k_1_ (min^−1^)	0.142	-	K_L_ (L/mg)	1.586	K_F_ (mg/g)	5.085	
				k_2_ (g/(mg min))	-	3.9 × 10^−3^	R^2^	1.000	R^2^	0.723	
				R^2^	0.922	1.000					
MoS_2_/MMT	HT	Hg(II)	35	q_e_ (mg/g)	1119.9	1390.81	q_m_ (mg/g)	2055.9	n	0.759	[99]
				k_1_ (min^−1^)	5.426 × 10^−3^	-	K_L_ (L/mg)	0.030	K_F_ (mg/g)	3.204	
				k_2_ (g/(mg min))	-	3.840 × 10^−6^	R^2^	0.944	R^2^	0.838	
				R^2^	0.982	0.96089					
MoS_2_-clinoptilolite	Commercial	Pb(II)	25	q_e_ (mg/g)	1.317	50	q_m_ (mg/g)	3.45	n	2.048	[8]
				k_1_ (min^−1^)	6.17 × 10^−2^	-	K_L_ (L/mg)	0.38	K_F_ (mg/g)	1.527	
				k_2_ (g/(mg min))	-	2.0 × 10^−2^	R^2^	0.964	R^2^	0.837	
				R^2^	0.723	1.000					
MoS_2_/Fe_3_O_4_	HT	Pb(II)	25	q_e_ (mg/g)	119.1	199.3	q_m_ (mg/g)	263.6	n	61.62	[111]
				k_1_ (min^−1^)	0.011	-	K_L_ (L/mg)	0.1468	K_F_ (mg/g)	3.354	
				k_2_ (g/(mg min))	-	1.7 × 10^−4^	R^2^	1.000	R^2^	0.841	
				R^2^	0.919	0.922					
MoS_2_-kaolin	HT	Pb(II)	25	q_e_ (mg/g)	12.23	65.94	q_m_ (mg/g)	280.39	n	5.71	[98]
				k_1_ (min^−1^)	−0.086		K_L_ (L/mg)	2.93	K_F_ (mg/g)	105.43	
				k_2_ (g/(mg min))		0.015	R^2^	0.863	R^2^	0.790	
				R^2^	0.0484	0.9922					
MoS_2_/LDC	HT	Cr(IV)	25	q_e_ (mg/g)	7.65	204.08	q_m_ (mg/g)	306.85	n	8.333	[112]
				k_1_ (min^−1^)	1.4 × 10^−2^	-	K_L_ (L/mg)	0.97	K_F_ (mg^1-n^ L^n^/g)	20.73	
				k_2_ (g/(mg min))	-	4 × 10^−4^	R^2^	0.86	R^2^	0.76	
				R^2^	0.68	0.999					
2D MoS_2_	HT	Ag(I)	25	q_e_ (mg/g)	392.73	410.24	q_m_ (mg/g)	813.01	n	3.62	[74]
				k_1_ (min^−1^)	0.921	^-^	K_L_ (L/mg)	0.12	K_F_ (mg/g)	206.51	
				k_2_ (g/(mg min))	-	3.0 × 10^−3^	R^2^	0.999	R^2^	0.911	
				R^2^	0.988	0.997					
chitosan-coated MoS_2_ 1:2	Precipitation method	Au(III)	30	q_e_ (mg/g)	1230.84	1338.55	q_m_ (mg/g)	3434.96	n	807.74	[28]
				k_1_ (min^−1^)	0.074	-	K_L_ (L/mg)	0.043	K_F_ (mg/g)	3.857	
				k_2_ (g/(mg min))	-	6.45 × 10^−5^	R^2^	0.991	R^2^	0.938	
				R^2^	0.724	0.901					
**Organic Contaminants**											
MoS_2_	HT	Bisphenol A	25	q_e_ (mg/g)	28.57	30.25	q_m_ (mg/g)		n		[83]
				k_1_ (min^−1^)	-	-	K_L_ (L/mg)		K_F_ (mg/g)		
				k_2_ (g/(mg min))	-	-	R^2^		R^2^		
				R^2^	0.901	0.998					
rGO/MoS_2_	HT	Ofloxacin		q_e_ (mg/g)	-	43.104	q_m_ (mg/g)	37.31	n	3.84	[7]
				k_1_ (min^−1^)	-	-	K_L_ (L/mg)	0.46	K_F_ (mg/g)	15.07	
				k_2_ (g/(mg min))	-	2.69 × 10^−4^	R^2^	0.989	R^2^	0.845	
				R^2^	-	0.918					
UiO−66/MoS_2_	HT	Lomefloxanin		q_e_ (mg/g)	45.6	80.67	q_m_ (mg/g)	32.36	n	5.152	[113]
				k_1_ (min^−1^)	0.2434	^-^	K_L_ (L/mg)	0.28606	K_F_ (mg/(g(L/mg)^1/n^))	14.957	
				k_2_ (g/(mg min))	-	1.7 × 10^−4^	R^2^	0.786	R^2^	0.940	
				R^2^	0.974	0.973					
MoS_2_/MTT	HT	Atenolol	25	q_e_ (mg/g)	88.85	96.62	q_m_ (mg/g)	145.6	n	3.363	[114]
				k_1_ (min^−1^)	0.151	-	K_L_ (L/mg)	0.197	K_F_ (L/g)	41.38	
				k_2_ (g/(mg min))	-	2.364 × 10^−3^	R^2^	0.974	R^2^	0.859	
				R^2^	0.957	0.991					
MoS_2_/MTT	HT	Acebutolol	25	q_e_ (mg/g)	63.85	69.25	q_m_ (mg/g)	130.8	n	2.772	[114]
				k_1_ (min^−1^)	0.287	-	K_L_ (L/mg)	0.107	K_F_ (L/g)	25.42	
				k_2_ (g/(mg min))	-	5.617 × 10^−3^	R^2^	0.945	R^2^	0.831	
				R^2^	0.829	0.982					
h-MoS_2_	HT	MO	25	q_e_ (mg/g)	12.86	42.44	q_m_ (mg/g)	38.11	n	1.897	[91]
				k_1_ (min^−1^)	0.518	-	K_L_ (L/mg)	0.285	K_F_ (mg/(g(L/mg)^1/n^))	5.342	
				k_2_ (g/(mg min))	-	9.35 × 10^−3^	R^2^	0.952	R^2^	0.992	
				R^2^	0.848	0.999					

h-MoS_2_—hollow MoS_2_ microspheres; HT—hydrothermal method; k_1_—rate constant of pseudo-first-order kinetic model; k_2_—rate constant of pseudo-second-order kinetic model; K_F_–Freundlich constant; K_L—_Langmuir constant; LDC—lignin-derived carbon; MMT—montmorillonite; MO–methyl orange; MoS_2_CAs—MoS_2_ composite aerogel; n–heterogeneity factor; PFO—pseudo-first-order kinetic model; PSO—pseudo-second-order kinetic model; q_e_—adsorption capacity at equilibrium; q_m_—maximum adsorption capacity; rGO—reduced graphene oxide; W-DR-N- MoS_2—_widened defect-rich nano MoS_2_.

**Table 2:** Characteristics of MoS_2_-based catalysts and the respective photocatalytic performance (in optimized conditions).

MoS_2_-Based Catalyst	Contaminant	MoS_2_ Production Method	Band Gap (eV)	CSA (m^2^/g)	Irradiation	Irradiation Time (min)	Removal Rate	PFO Kinetics k (min^−1^)	Ref.
**Dyes**									
α-Fe_2_O_3_/MoS_2_QDs	MB	HT	2.22	5.852	Vis (400–700 nm)	1 120	83% 91%	1st min 0.892 > 28 min 0.0049	[92]
MoS_2_/ZnO	MB	HT	-	14.785	Vis	40	97%	0.0701	[121]
5 wt% MoS_2_/BiFeO_3_/Ag_3_PO_4_	MB	C	2.07	7.705	UV-Vis	5	97%	0.625	[119]
ZnO/MoS_2_ 8 h	MB	LPE	1.77	-	UV-Vis	37.5	Vis-85% UV-78%	-	[124]
Ni foam with ZnO/MoS_2_	MB	HT	-	4.85	UV-Vis	50	72%	-	[120]
1.9% MoS_2_/g-C_3_N_4_/PAN	MB	HT	2.76	15.6	Vis (>420 nm)	120	85%	-	[125]
MoS_2_/Fe_3_O_4_ (MF-17)	MB	HT	-	4,5	Vis	120	98%	-	[25]
CM12 (Cu_2_O/MoS_2_)	MB	HT	Cu_2_O—2.15 MoS_2_—1.76	-	Vis (>420 nm)	30	90%	0.084	[126]
MoS_2_-TiO_2_	MO	HT	2.76	103.5	-	60	86%	0.016	[127]
CoO/meso-CN/MoS_2_ (1%) (S6)	MO	HT	2.82–2.86	37.64	VIS	60	84%	-	[118]
5 wt% MoS_2_/BiFeO_3_/Ag_3_PO_4_	MO	C	2.07	7.704	UV-Vis	5	98%	0.376	[119]
g-C_3_N_4_/MoS_2_/TiO_2_ (CMT5)	MO	HT	2.64	97.5	Vis (400–700 nm)	60	98%	0.061	[128]
Ni foam with ZnO/MoS_2_	MO	HT	-	4.85	UV-vis	10	93%	0.059	[122]
5% MoS_2_/ZnO	RhB	HT	3.18	-	UV	50	95%	0.057	[119]
5 wt% MoS_2_/BiFeO_3_/Ag_3_PO_4_	RhB	C	2.07	7.704	UV-Vis	5	97%	0.676	[119]
Ag/MoS_2_/CC	RhB	HT	-	27	Vis	20 40	90% 99%	72.1 × 10^−3^	[129]
CTAB-MoS_2_-P25	RhB	HT	2.06	-	-	120	91%	-	[130]
Ni foam with ZnO/MoS_2_	RhB	HT	-	4.85	UV-vis	80	8%	-	[120]
1.9% MoS_2_/g-C_3_N_4_/PAN	RhB	HT	2.76	15.6	Vis (>420 nm)	120	48%	0.006	[125]
BiOI/MoS_2_ (BMS-8)	RhB	HT	BiOI—1.42 MoS_2_—1.73	30.76	Vis (>420 nm)	90	100%	0.023	[3]
MoS_2_/Fe_3_O_4_ (MF-17)	RhB	HT	-	4,5	Vis	120	96%	-	[25]
g-C_3_N_4_/MoS_2_/GO (AT3G15)	RhB	HT	2.05	-	Vis	60	99%	-	[131]
5wt% MoS_2_/BiFeO_3_/Ag_3_PO_4_	Acrid red 18	C	2.07	7.7045	UV-Vis	7	98%	0.484	[119]
CoO/meso-CN/MoS_2_(1%) (S6)	Methyl red	HT	2.82–2.86	37.64	Vis	60	96%	0.072	[118]
CoO/meso-CN/MoS_2_(1%) (S6)	Congo red	HT	2.82–2.86	37.64	Vis	60	95%	-	[118]
g-C_3_N_4_/MoS_2_/TiO_2_ (CMT5)	4-Nitrophenol	HT	2.64	97.5	Vis (400–700 nm))	60	87%	-	[128]
MoS_2_-ZnS	Crystal violet	HT	-	-	Vis	40	99%	41.26 × 10^−3^	[132]
Ni foam with ZnO/MoS_2_	Congo red	HT	-	4.85	UV-Vis	80	77%		[120]
MoS_2_/g-C_3_N_4_/TiO_2_	Malachite green	HT	2.42	-	Vis	60	86%	0.045	[133]
**Antibiotics**									
MoS_2_/BiOBr/CF	TC	HT	MoS_2—_1.81 BiOBr—2.88	-	Vis	120	89%	-	[134]
Fe_3_O_4_/MoS_2_/BiVO_4_ (FMB3)	TC	HT	MoS_2_—1.56 BiVO_4_—2.44	-	Vis (>420 nm)	120	86%	0.01576	[135]
5 wt% MoS_2_/BiFeO_3_/Ag_3_PO_4_	TC	C	2.07	7.704	UV-Vis	120	90%	-	[119]
MoS_2_/Z-50	TC	HT	-	17.32	Vis (>420 nm)	120	97%	-	[136]
20 wt% MoS_2_/BEuCN	TC	HT	2.85	44.12	Vis	50	99%	0.087	[122]
MoS_2/_TiO_2_/graphene (MTG-48)	TC	HT	3.17	58.6	UV-Vis (300–750 nm)	60	92%	0.05	[137]
MoS_2_/ZnO QDs	TC	HT	-	-	Vis	80	96%	0.01	[138]
CdS/MoS_2_/ZnO(CMZ)	Ofloxacin	chemical co-precipitation method	CdS—2.145 MoS_2_—2.015 ZnO—2.981	-	Vis (>420 nm)	90	89%	0.024	[139]
UiO-66/MoS_2_ (UMS-0.15)	Lomefloxacin	HT	-	37.176	Vis	90	87%	-	[113]
**Other organic compounds**									
MXene-Ti_3_C_2_/MoS_2_ (MT-4)	Ranitidine	HT	1.59	11.93	Vis	60	88%	-	[140]
rGO10%/ZnO20%/MoS_2_	Aniline	HT	2.24	-	Vis	120	100%	-	[141]
MoS_2_-TiO_2_	4-Nitrophenol	HT	2.76	103.5	Vis	60	97%	0.024	[127]
MoS_2_/CuO-25%	2-MBT	HT	MoS_2_—1.52 CuO—2.16	47	Vis	120	96%	-	[142]
Cu_2_S-1.0%MoS_2_	Phenol	C	1.42	-	Vis	90	90%	-	[143]
g-C_3_N_4_/MoS_2_/GO (AT3G15)	4-CP	HT	2.05	-	Vis	60	89%	-	[131]
5 wt% MoS_2_/BiFeO_3_/Ag_3_PO_4_	2,4-D	C	2.07	7.704	UV-Vis	180	90%	-	[119]
5 wt% MoS_2_/BiFeO_3_/Ag_3_PO_4_	Acephate	C	2.07	7.704	UV-Vis	60	85%	-	[119]
g-C_3_N_4_/MoS_2_−PANI	Bisphenol-A	HT	2.67	184.21	Vis	60	93%	0.040	[123]
**Metal ion species**									
MoS_2_/BiOBr/CF	Cr(VI)	HT	MoS_2_—1.81 BiOBr—2.88	-	Vis	120	85%	-	[134]
Ni foam with ZnO/MoS_2_	Cr(VI)	HT	-	4.85	UV-Vis	25	75%	-	[120]
MoS_2/_TiO_2_/graphene (MTG-48)	Pb(II)	HT	3.17	58.6	UV-Vis (300–750 nm)	60	88%	-	[137]

Note: 2,4-D—2,4-dichlorophenoxyacetic acid; 2-MBT—2-mercaptobenzothiazole; 4-CP—4-chlorophenol; C—commercial; CC—carbon cloth; CF—carbon fiber; CTAB- cetyltrimethyl ammonium bromide; CSA—catalyst-specific area; HT—hydrothermal method; LPE—liquid-phase exfoliation method; MB—methylene blue; MO—methyl orange; PAN—polyacrylonitrile; PANI—polyaniline; PFO—pseudo-first order; QDs—quantum dots; RhB—rhodamine B; Z-50—zeolite; TC—tetracycline.

**Table 3:** Photodegradation rate and mineralization evaluation on photocatalytic studies, with MoS_2_-based catalysts.

MoS_2_-Based Catalyst	Contaminant	Irradiation Time (min)	Photodegradation/Removal Rate	Mineralization Evaluation	Ref.
5 wt % MoS_2_/BiFeO_3_/Ag_3_PO_4_	MB	5	97%	>85%	[119]
CoO/meso-CN/MoS_2_(1%) (S6)	MO	60	84%	54%	[118]
5 wt % MoS_2_/BiFeO_3_/Ag_3_PO_4_	MO	5	98%	> 85%	[119]
g-C_3_N_4_/MoS_2_/TiO_2_ (CMT5)	MO	60	98%	91%	[128]
5 wt % MoS_2_/BiFeO_3_/Ag_3_PO_4_	RhB	5	97%	>85%	[119]
BiOI/MoS_2_ (BMS-8)	RhB	90	100%	78% *	[3]
g-C_3_N_4_/MoS_2_/GO (AT3G15)	RhB	60	99%	85%	[131]
5 wt % MoS_2_/BiFeO_3_/Ag_3_PO_4_	Acrid red 18	7	98%	>85%	[119]
CoO/meso-CN/MoS_2_(1%) (S6)	Methyl red	60	96%	80%	[118]
CoO/meso-CN/MoS_2_(1%) (S6)	Congo red	60	95%	71%	[118]
MoS2/BiOBr/CF	TC	120	92%	55% **	[134]
5 wt % MoS_2_/BiFeO_3_/Ag_3_PO_4_	TC	120	90%	91%	[119]
MoS_2/_TiO_2_/graphene (MTG-48)	TC	60	92%	33.8%	[137]
MXene-Ti_3_C_2_/MoS_2_ (MT-4)	Ranitidine	60	88%	74%	[140]
5 wt % MoS_2_/BiFeO_3_/Ag_3_PO_4_	2,4-D	180	90%	87%	[119]
5 wt % MoS_2_/BiFeO_3_/Ag_3_PO_4_	Acephate	60	85%	93%	[119]

Note: 2,4-D—2,4-dichlorophenoxyacetic acid; CF—carbon fiber; MB—methylene blue; MO—methyl orange; RhB—rhodamine B; TC—tetracycline; * TOC after 60 min of irradiation; ** TOC after 7 h of irradiation.

**Table 4:** Adsorption and photocatalytic performance of MoS_2_-based nanocomposites and a comparison with bare MoS_2_.

**Adsorption**									
**Composite**	**Contaminant**	**Specific Area (m^2^/g)**	**Contact Time (min)**	**Comparative Adsorption Parameters**					**Ref.**
W-DR-N-MoS_2_	Hg(II)	-	-	Interlaying spacing	9.42 Å	Maximum adsorption capacity (q_max_) (mg g^−1^)		2562.8	[97]
MoS_2_	Hg(II)	-	-		6.15 Å			35.5	
MoS_2_/MTT	Hg(II)	-	-	Adsorption capacity in equilibrium, according to the pseudo-first-order kinetic model (mg g^−1^)				1119.94	[99]
MoS_2_	Hg(II)	-	-					936.62	
MoS_2_-kaolin	Pb(II)	14.56	10	Adsorption capacity (mg g^−1^)		55.10	Removal rate	89%	[98]
MoS_2_	Pb(II)	13.08	10			35.68		54%	
MoS_2_/MTT	Atenolol	-	150	Adsorption capacity (mg g^−1^)			132.08		[114]
MoS_2_	Atenolol	-	150				74.23		
MoS_2_/MTT	Acebutolol	-	150	Adsorption capacity (mg g^−1^)			113.82		[114]
MoS_2_	Acebutolol	-	150				36.05		
**Photocatalysis**									
**Catalyst**	**Contaminant**	**Band Gap (eV)**		**Specific Area (m^2^/g)**	**Irradiation Time (min)**	**Removal Rate (%)**	**PFO Kinetics** **k (min^−1^)**		**Ref.**
MZO (MoS_2_/ZnO)	MB	-		14.785	40	97	0.070		[121]
MoS_2_	MB	-		3.795	40	44	0.010		
MoS_2_/Fe_3_O_4_ (MF-17)	MB	-		4.5	120	98	-		[25]
MoS_2_	MB	-		9.0	120	92	-		
MoS_2_-TiO_2_	MO	2.76		103.5	60	86	0.016		[127]
MoS_2_	MO	3.26		88.5	60	53	0.008		
BiOI/MoS_2_ (BMS-8)	RhB	BiOI 1.42 MoS_2_ 1.73		30.76	90	100	0.023		[3]
MoS_2_	RhB	MoS_2_ 1.73		49.59	90	60	0.009		
MoS_2_/Fe_3_O_4_ (MF-17)	RhB	-		4.5	120	96	-		[25]
MoS_2_	RhB	-		9.0	120	82	-		
MoS_2/_TiO_2_/graphene (MTG-48)	TC	3.17		58.6	60	92	0.05		[137]
MoS_2_	TC	2.66		29.3	60	54	0.018		
MoS_2_-ZnS	Crystal violet	-		-	40	98.5	41.26 × 10^−3^		[132]
MoS_2_	Crystal violet	-		-	40	60	24.23 × 10^−3^		
MoS_2_-TiO_2_	4-Nitrophenol	2.76		103.5	60	97	0.024		[127]
MoS_2_	4-Nitrophenol	3.26		88.5	60	59	0.009		
UiO-66/MoS_2_ (UMS-0.15)	Lomefloxacin	-		37.176	90	87	37.176		[113]
MoS_2_	Lomefloxacin	-		7.775	90	38.2	7.775		
MoS_2_/CuO-25%	2-MBT	MoS_2_ 1.52 CuO 2.16		47	120	96	-		[142]
MoS_2_	2-MBT	1.52		14.76	120	22	-		

Note: 2-MBT—2,4-D—2,4-dichlorophenoxyacetic acid; MB—methylene blue; MO—methyl orange; MMT—montmorillonite; RhB—rhodamine B; TC—tetracycline; W-DR-N-MoS_2_—Widened Defect Rich Nano MoS_2_.

**Table 5:** Reuse of MoS_2_-based sorbents and photocatalysts.

**Adsorption**						
**MoS_2_-Based Composite**	**Contaminant**	**Contact Time (min)**	**Removal** **1st Cycle (%)**	**Number of Cycles**	**Removal after Last Cycle (%)**	**Ref.**
Au/Fe_3_O_4_/MoS_2_CAs	Hg(II)	30	95–100	10	>95	[109]
MoS_2_/Fe_3_O_4_	Hg(II)	-	100–95	5	identical	[111]
MoS_2_-kaolin	Hg(II)	50	99	5	77	[98]
MoS_2_-rGO	Hg(II)	7	100	6	85–90	[110]
MoS_2_/MTT	Hg(II)	-	100	4	85.2	[99]
MoS_2_/Fe_3_O_4_	Pb(II)	-	100–95	5	identical	[111]
MoS_2_/LDC	Cr(VI)	30	99	4	90	[112]
chitosan-coated MoS_2_ 1:2	Au(III)	-	98.9	4	86.4	[28]
MoS_2_-rGO	Ofloxacin	-	92	8	4	[7]
**Photocatalysis**						
**MoS_2_-Based Catalyst**	**Contaminant**	**Irradiation Time (min)**	**Removal** **1st Cycle (%)**	**Number of Cycles**	**Removal after Last Cycle (%)**	**Ref.**
α-Fe_2_O_3_/MoS_2_QDs	MB	1 120	83 91	3	1min 65 120 min 75	[92]
MZO (MoS_2_/ZnO)	MB	40	97	5	89.10	[121]
ZnO/MoS_2_ 8 h	MB	37.5	85	5	65	[124]
CM12 (Cu_2_O/MoS_2_)	MO	30	90	5	40	[126]
CoO/meso-CN/MoS_2_(1%) (S6)	MO	60	84	5	80	[118]
Ni foam with ZnO/MoS_2_	MO	10	92.7	4	86.9	[120]
5% MoS_2_/ZnO	RhB	50	95	5	87	[93]
CTAB-MoS_2_-P25	RhB	120	91	4	76	[130]
1.9% MoS_2_/g-C_3_N_4_/PAN	RhB	120	48	4	43	[125]
BiOI/MoS_2_ (BMS-8)	RhB	90	100	6	>90	[3]
g-C_3_N_4_/MoS_2_/GO (AT3G15)	RhB	60	99	5	92	[131]
CoO/meso-CN/MoS_2_(1%) (S6)	Methyl red	60	96	5	90	[118]
CoO/meso-CN/MoS_2_(1%) (S6)	Congo red	60	95	5	88	[118]
MoS_2_-ZnS	Crystal violet	40	98.5	4	90.5	[132]
MoS_2_/g-C_3_N_4_/TiO_2_	Malachite green	60	86	4	70	[133]
MoS_2_/BiOBr/CF	TC	120	89.0	4	80.7	[134]
Fe_3_O_4_/MoS_2_/BiVO_4_ (FMB3)	TC	120	86.1	5	>80	[135]
MoS_2_/Z-50	TC	120	96.8	5	87.2	[136]
20 wt% MS/BEuCN	TC	50	99	3	identical	[120]
MoS_2/_TiO_2_/graphene (MTG-48)	TC	60	92	5	85	[137]
MoS_2_/ZnO QDs	TC	80	96	5	89	[138]
CdS/MoS_2_/ZnO(CMZ)	Ofloxacin	90	89	4	70–75	[139]
UiO-66/MoS_2_ (UMS-0.15)	Lomefloxacin	90	87	4	79	[113]
MXene-Ti_3_C_2_/MoS_2_ (MT-4)	Ranitidine	60	88.4		80	[140]
RGO10%/ZnO20%/MoS_2_	Aniline	120	100	5	100	[141]
MoS_2_/CuO-25%	2-MBT	120	96	5	91	[142]
MoS_2_/BiOBr/CF	Cr(VI)	120	84.7	4	76.6	[134]
MoS_2/_TiO_2_/graphene (MTG-48)	Pb(II)	60	88	6	80	[137]

Note: 2-MBT—2-mercaptobenzothiazole; CF—carbon fiber; CTAB—cetyltrimethyl ammonium bromide; LDC—lignin-derived carbon; MB—methylene blue; MO—methyl orange; MTT—montmorillonite; QDs—quantum dots; rGO—reduced graphene oxide; RhB—rhodamine B; TC—tetracycline.

## Data Availability

Not applicable.
